# NMR Structure of the Human Prion Protein with the Pathological Q212P Mutation Reveals Unique Structural Features

**DOI:** 10.1371/journal.pone.0011715

**Published:** 2010-07-22

**Authors:** Gregor Ilc, Gabriele Giachin, Mariusz Jaremko, Łukasz Jaremko, Federico Benetti, Janez Plavec, Igor Zhukov, Giuseppe Legname

**Affiliations:** 1 Slovenian NMR Centre, National Institute of Chemistry, Ljubljana, Slovenia; 2 Laboratory of Prion Biology, Neurobiology Sector, Scuola Internazionale Superiore di Studi Avanzati (SISSA), Trieste, Italy; 3 Laboratory of Biological NMR, Institute of Biochemistry and Biophysics, Polish Academy of Sciences, Warsaw, Poland; 4 Faculty of Chemistry, Warsaw University, Warsaw, Poland; 5 SISSA Unit, Italian Institute of Technology, Trieste, Italy; 6 Faculty of Chemistry and Chemical Technology, University of Ljubljana, Ljubljana, Slovenia; 7 ELETTRA Laboratory, Sincrotrone Trieste S.C.p.A., Trieste, Italy; University of Kent, United Kingdom

## Abstract

Prion diseases are fatal neurodegenerative disorders caused by an aberrant accumulation of the misfolded cellular prion protein (PrP^C^) conformer, denoted as infectious scrapie isoform or PrP^Sc^. In inherited human prion diseases, mutations in the open reading frame of the PrP gene (*PRNP*) are hypothesized to favor spontaneous generation of PrP^Sc^ in specific brain regions leading to neuronal cell degeneration and death. Here, we describe the NMR solution structure of the truncated recombinant human PrP from residue 90 to 231 carrying the Q212P mutation, which is believed to cause Gerstmann-Sträussler-Scheinker (GSS) syndrome, a familial prion disease. The secondary structure of the Q212P mutant consists of a flexible disordered tail (residues 90–124) and a globular domain (residues 125–231). The substitution of a glutamine by a proline at the position 212 introduces novel structural differences in comparison to the known wild-type PrP structures. The most remarkable differences involve the C-terminal end of the protein and the β_2_–α_2_ loop region. This structure might provide new insights into the early events of conformational transition of PrP^C^ into PrP^Sc^. Indeed, the spontaneous formation of prions in familial cases might be due to the disruptions of the hydrophobic core consisting of β_2_–α_2_ loop and α_3_ helix.

## Introduction

Transmissible spongiform encephalopathies (TSE), or prion diseases, are a rare group of neuropathies characterized by a spongiform neurodegeneration of the brain caused by prions. Amyloid deposits may parallel the pathology and are mainly composed by the abnormal, misfolded form of the cellular prion protein (PrP^C^) denominated PrP^Sc^. The unique etiology of this group of maladies can be sporadic, inherited and iatrogenic [1]. These disorders include Creutzfeldt–Jakob disease (CJD), Gerstmann-Sträussler-Scheinker (GSS) syndrome, Fatal Familial Insomnia (FFI) and kuru in humans, bovine spongiform encephalopathy in cattle, scrapie in sheep, and chronic wasting disease in elk, deer and moose.

The human (Hu) PrP^C^ is a 209 residues glycoprotein, attached by a C-terminal glycosylphosphatidylinositol to the outer leaflet of plasma membrane of the cell and is highly conserved among mammals. Although several processes in the nervous system are influenced by PrP^C^, its physiological function still remains elusive [2]. According to the “protein-only hypothesis”, in prion disease PrP^C^ is converted into the abnormal type by a conversion process whereby most α-helix motives are replaced by β-sheet secondary structures [3]. The PrP^C^ to PrP^Sc^ conversion leads to altered biochemical properties, such as resistance to limited proteolysis and insolubility in non-denaturant detergents [4], [5].

Although the molecular mechanisms leading to the disease are still controversial, many evidences suggest that the generation of prion disease is dependent only on PrP^C^. Mice devoid of PrP^C^ are resistant to scrapie, and the reintroduction of the PrP gene (*Prnp* in mice) restores TSE susceptibility [6], [7]. Moreover, amyloid PrP fibrils generated *in vitro* induced prion disease in transgenic mice overexpressing PrP, which was subsequently transmissible to wild-type (WT) mice [8], [9].

One of the strongest arguments supporting the “protein-only hypothesis” is the link between inherited prion diseases and mutations in the *PRNP* gene. Several point mutations leading to familial CJD, GSS or FFI have been identified in the open reading frame of the *PRNP* gene [10]. Transgenic mice carrying pathological PrP mutations develop a spectrum of neurological diseases sharing some features with TSE [11], [12], [13], [14]. Our understanding of the mechanisms by which mutations induce the disease still remains limited. Mutations may increase the likelihood of misfolding by the thermodynamic destabilization of PrP^C^ [15], [16], [17], [18]. PrP mutants may escape quality control cellular pathway and accumulate inside the cell [19], [20], [21], [22]. In addition, mutations may change surface properties promoting an abnormal interaction between PrP^C^ and other not yet identified interactors [23], [24].

NMR studies of recombinant HuPrP(90–231) reveal a structure containing a short flexible chain at the C-terminal, a globular domain with three alpha helices (α_1_, α_2_ and α_3_), a short anti-parallel beta sheet (β_1_ and β_2_), and an unstructured N-terminal tail [25], [26]. Up to date, there is no evidence showing that a pathological point mutation may cause substantial structural differences in the PrP fold. Indeed, solution structures of some pathogenic HuPrP mutants exhibit conformations similar to the WT protein [27], [28].

To provide new clues on the role of pathological point mutations on PrP structure, in this study we determined and examined a high-resolution 3D structure of the truncated recombinant HuPrP(90–231) containing the pathological Q212P mutation. This mutation is responsible for a GSS syndrome characterized by mild amyloid PrP deposition in patients [29], [30]. Subsequently, we compared our structural findings with the already resolved NMR structures of HuPrP carrying the CJD-related E200K [28] and the artificial R220K mutations [31]. The substitution of a glutamine by a proline at the position 212 revealed novel and unique structural differences in comparison to the known structures of either human or other mammalian PrP^C^ [32], [33], [34], [35], [36].

## Results

### Location and nature of mutation at codon 212

The Q212P mutation in PrP^C^ is associated with GSS, a slowly progressive hereditary autosomal dominant disease. Interestingly, neuro-pathological examination of CNS of patients with this disorder showed mild amyloid PrP deposition, and the disease presented reduced penetrance among relatives [29], [30]. The hallmark of the GSS neuropathology is the encephalo(myelo)pathy with multi-centric PrP plaques [37]. Epidemiologic data of GSS are difficult to estimate, but figures within the range of 1–10/100,000,000 are quoted [38], [39]. Amongst the inherited prion diseases, Q212P is a very rare point mutation: only one family case carrying this mutation has been described. Gln212 is highly conserved among mammalian and non-mammalian PrP [40]. This amino acid residue is located in the middle of α_3_ helix in close proximity to the disulfide bond Cys179 – Cys214 and forms a hydrogen bond with Thr216 [41].

### Sequence-specific resonance assignment of HuPrP (90–231, M129, Q212P)

**Figure 1:**
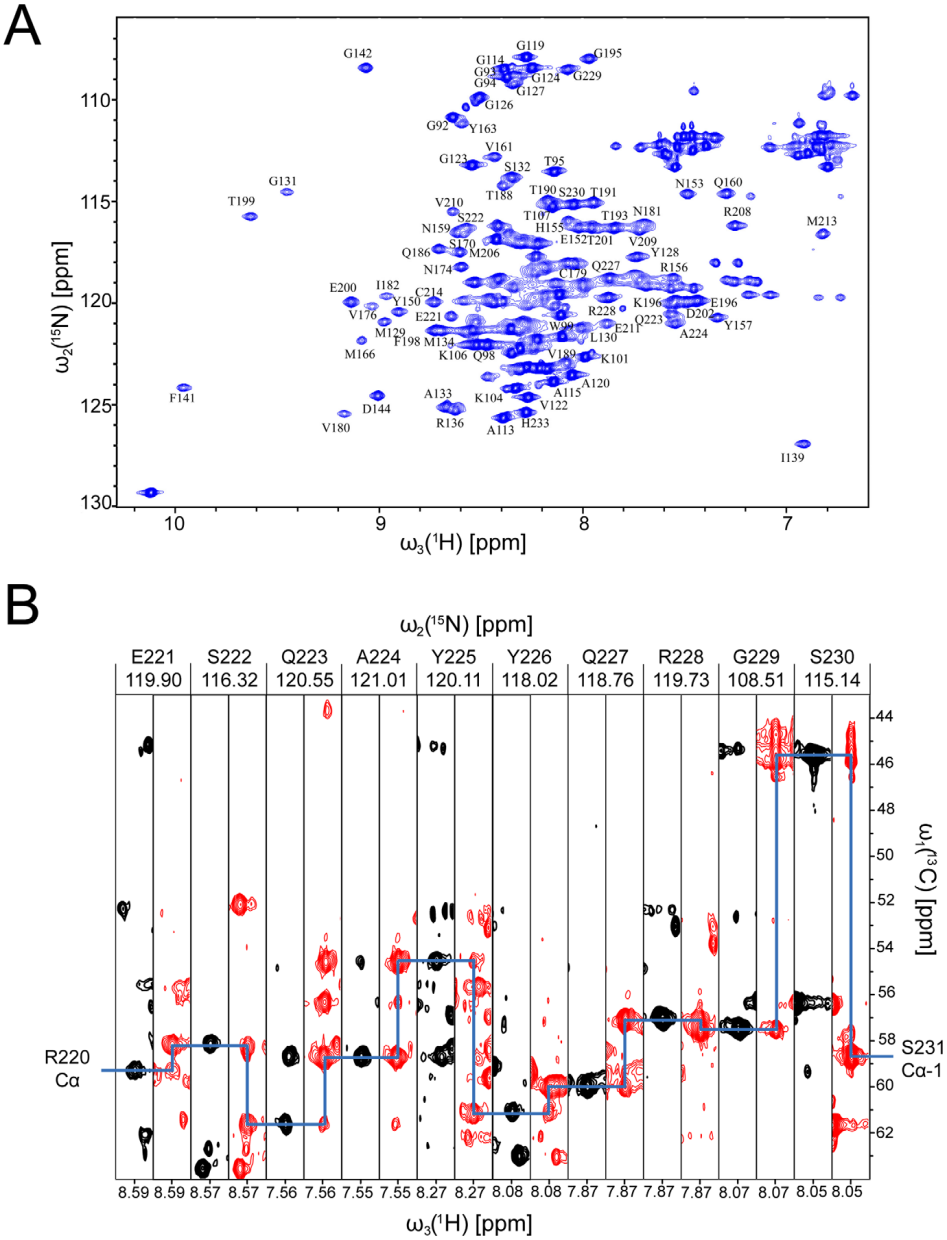
NMR spectra and assignment of HuPrP(90–231, M129, Q212P). (A) ^1^H-^15^N HSQC spectrum with one letter amino acid code. (B) Backbone assignment for the part that shows unique structural features. Strips for residues 221-230 from 3D CA(CO)NH and HNCA experiments with cross-peaks in black from ^13^C_α_(*i-1*) nuclei in CA(CO)NH experiment and in red corresponding to ^13^C_α_(*i-1*) and ^13^C_α_(*i*) nuclei revealed by HNCA experiment. Blue line indicates sequential walk.

^1^H-^15^N HSQC spectrum of ^13^C, ^15^N double labeled mutant demonstrated good dispersion of amide signals (Figure 1A). Cross-peaks for all residues could be identified with the exception of Arg164, Tyr169, Asn171, Phe175 and Gln217, which were not observed due to line broadening caused by exchange processes (*see below*). The sequence-specific assignment was achieved with the use of standard triple resonance HNCA, HN(CO)CA, HNCACB and CBCA(CO)NH NMR experiments. Strips from 3D CA(CO)NH and HNCA experiments in Figure 1B illustrate sequential walk in the region from Glu221 to Ser230. The assignment was additionally confirmed by analysis of sequential and medium-range NOEs in 3D ^15^N-edited NOESY-HSQC experiment. ^1^H and ^13^C resonances of side chains were assigned by analyses of 3D (H)CCH-TOCSY and ^13^C-edited NOESY-HSQC spectra. Final level of completeness of ^1^H, ^13^C and ^15^N resonance assignment was very high (95.1%). Chemical shifts were deposited in BioMagnetic Resonance data Bank (BMRB, accession code 16743).

The C_β_ chemical shifts of Cys179 and Cys214 (40.3 and 41.5 ppm, respectively) confirmed the presence of the disulfide bond. Conformations of peptide bonds in X-Pro fragments were deduced from chemical shifts of C_β_ and C_γ_. All peptide bonds were shown to adopt *trans* conformation, which was additionally confirmed by the corresponding cross-peaks in 3D ^13^C-edited NOESY-HSQC spectra.

### Three-dimensional structure of Q212P mutant

**Figure 2:**
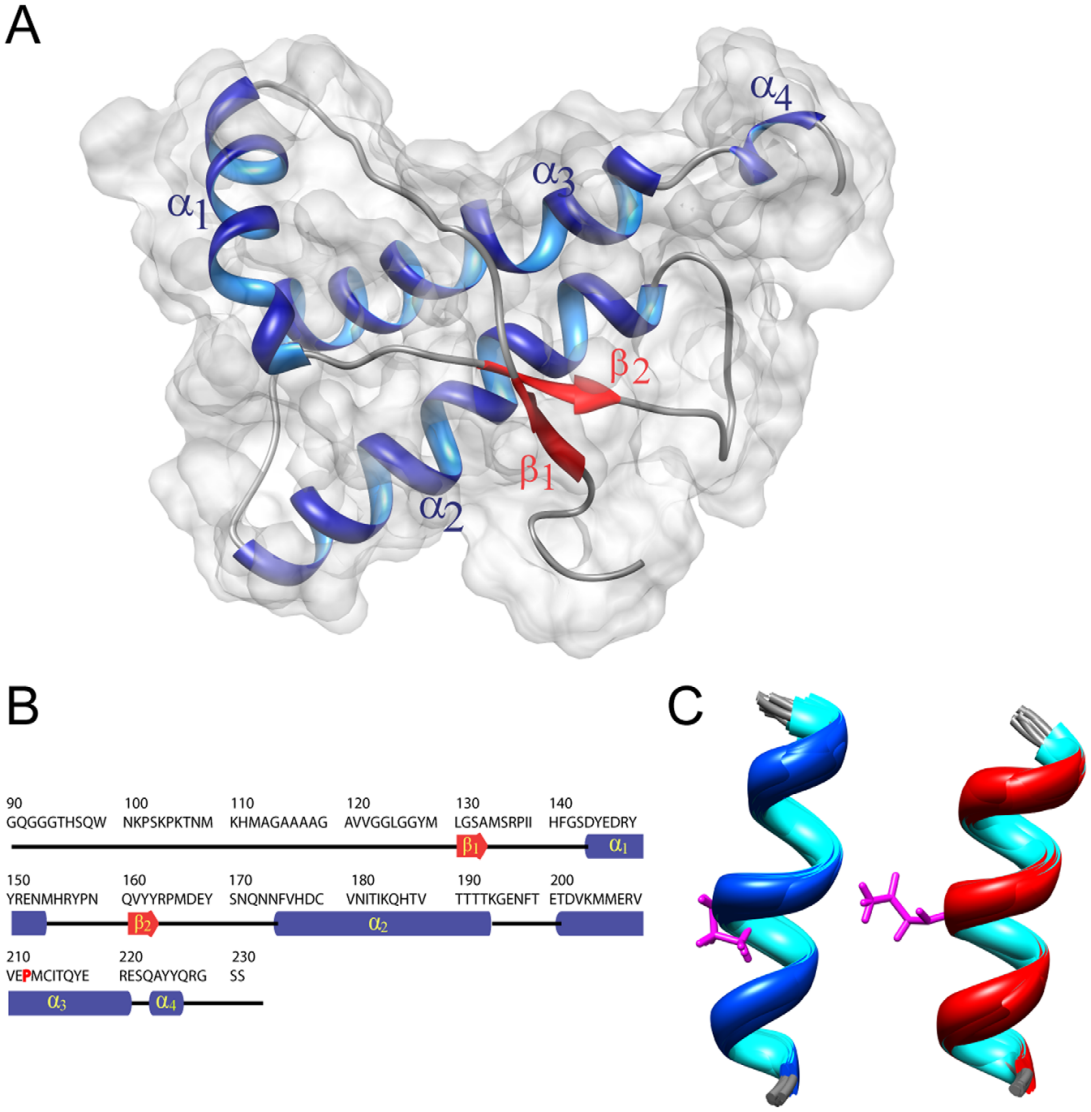
High-resolution structure of HuPrP(90–231, M129, Q212P). (A) Cartoon representation of the lowest energy structure on the van der Waals surface. (B) Sequence of HuPrP(90–231, M129, Q212P) protein. The elements of secondary structure are shown. (C) Structural details of α_3_ helix in the family of 20 lowest energy structures from Met205 to Arg220 in the Q212P mutant (left, pdb id 2KUN) and WT HuPrP^C^ (right, pdb id 1QM1) [25]. Residues Pro212 and Gln212 are presented in pink. The r.m.s.d. for backbone atoms in residues between Met205 and Arg220 in both ensembles is 0.7 Å.

The high number of NOE restrains, together with completeness of resonance assignments, allowed us to determine the structure of Q212P mutant with high resolution (Figure 2 and Table 1). The three-dimensional structure of the Q212P mutant consists of a well-defined globular domain and highly disordered N-terminal tail. The C-terminal globular domain (residues 125–231) is composed of a short anti-parallel β-sheet and four α helices.

**Table 1:** NMR restrains and structural statistics for the ensemble of 20 lowest energy structures of HuPrP(90-231, M129, Q212P) protein.

NOE upper distance limits^a^	2205
Intra-residue & sequential (|*i-j*|≤1)	1244
Medium-range (|*i-j*|<5)	491
Long-range (|*i-j*|>5)	470
Torsion angle constraints^a^	
backbone (φ/ψ)	188
r.m.s.d. from idealized covalent geometry	
bonds (Å)	0.00125±0.00002
angles (deg)	0.249±0.002
impropers (deg)	0.141±0.003
r.m.s.d. to the mean coordinates (Å)	
ordered backbone atoms (125..226)	1.01±0.34
Ordered heavy atoms (125..226)	1.45±0.31
Ramachandran plot (%)b,d	
Residues in most favored regions (%)	92.1
Residues in additional allowed regions (%)	7.2
Residues in generously allowed regions (%)	0.4
Residues in disallowed regions	0.3
Structure Z-scoresc,d	
1^st^ generation packing quality	−3.198±0.230
2^nd^ generation packing quality	−2.709±0.252
Ramachandran plot apperance	−2.584±0.457
Chi-1/chi-2 rotamer quality	−4.098±0.386
Backbone conformation	−2.872±0.444
RMS Z-scoresc,d	
Bond lenghts	1.084±0.045
Bond angles	0.539±0.038
Omega angle restrains	0.769±0.042
Side chain planarity	1.125±0.200
Improper dihedral distribution	0.886±0.067
Inside/Outside distribution	1.008±0.008

a None of the 20 structures exhibits distance violation over 0.2 Å and torsion angle violation over 5°.

b Ensemble of structures was analyzed by PROCHECK-NMR (version 3.4) program [57].

c Ensemble of structures was validated and analyzed using WhatIF program [58].

d Validation procedure was performed on the structured part from residues 125 to 231.

**Figure 3:**
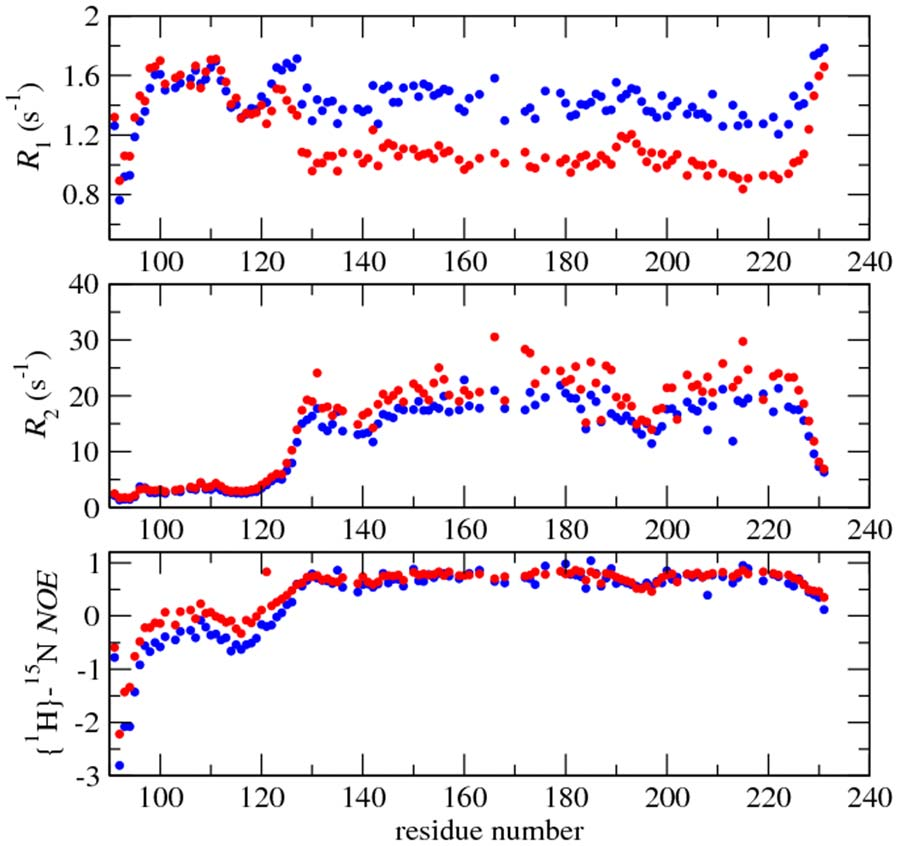
^15^N relaxation rates for HuPrP(90–231, M129, Q212P) protein at 298K. ^15^N longitudinal (*R*_1_), transverse (*R*_2_) relaxation rates and heteronuclear {^1^H}-^15^N NOE data acquired at 11.7 T and 16.4 T shown in blue and red, respectively.

**Figure 4:**
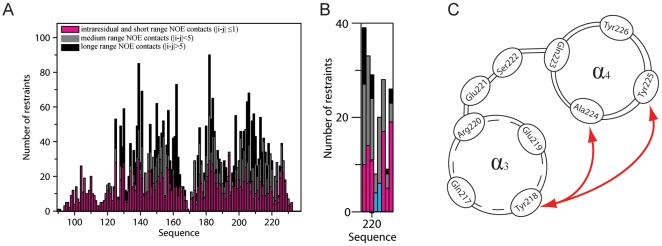
Distance restrains per residue. (A) Type of NOE used in structure calculations of HuPrP(90–231, M129, Q212P) protein. (B) Enlarged region between Tyr218 and Tyr225. Glu221 and Ser222 do not exhibit any long-range NOE contacts. (C) Schematic presentation of long-range NOE contacts (|i-j|>5) between residues in helices a_3_ and a_4_. For clarity, only inter-helical NOE contacts are shown.

A detailed analysis of the structures ensemble, and comparison to the known structures of PrP^C^ proteins from human and other mammals, indicated that the substitution of the glutamine by a proline at the codon 212 resulted in minor if any local structural changes. The comparison of structural details of α_3_ helices in Q212P mutant and WT protein showed small differences with r.m.s.d. of 0.7 Å for backbone atoms between Met205 and Arg220 (Figure 2C). However, the mutation carried notable differences in the overall structure of C-terminal domain. In particular, while α_3_ helix is well ordered from residue Glu200 to Arg220, it does not exhibit properties of a common helical conformation for the subsequent two residues in primary sequence of Q212P mutant. Glu221 and Ser222 interrupt the regular structure of α_3_ helix (Figure 2A). In fact, the region between Glu200 and Tyr226 consists of two helices that are annotated by us as α_3_ and α_4_. The collected NMR relaxation data, together with the low number of NOE restrains for Glu221 and Ser222, additionally confirmed an increased mobility, and the disruption of helical conformation at those positions (Figures 3, 4A and 4B). Medium-range NOE interactions proved the existence of one turn of a regular α helix (i.e. α_4_ helix) within the region from Gln223 to Tyr226.

The observations of long-range NOE contacts amongst residues Tyr218, Ala224 and Tyr225 in α_3_ and α_4_ helices demonstrate their spatial relationships in the calculated structures (Figure 4C). The α_4_ helix exhibits increased molecular mobility that is evidenced through reduced heteronuclear {^1^H}-^15^N NOE values in combination with increased ^15^N *R*_1_ and reduced ^15^N *R*_2_ relaxation rates (Figure 3).

### β_2_–α_2_ loop region exposes hydrophobic surface that is available for intermolecular interactions

**Figure 5:**
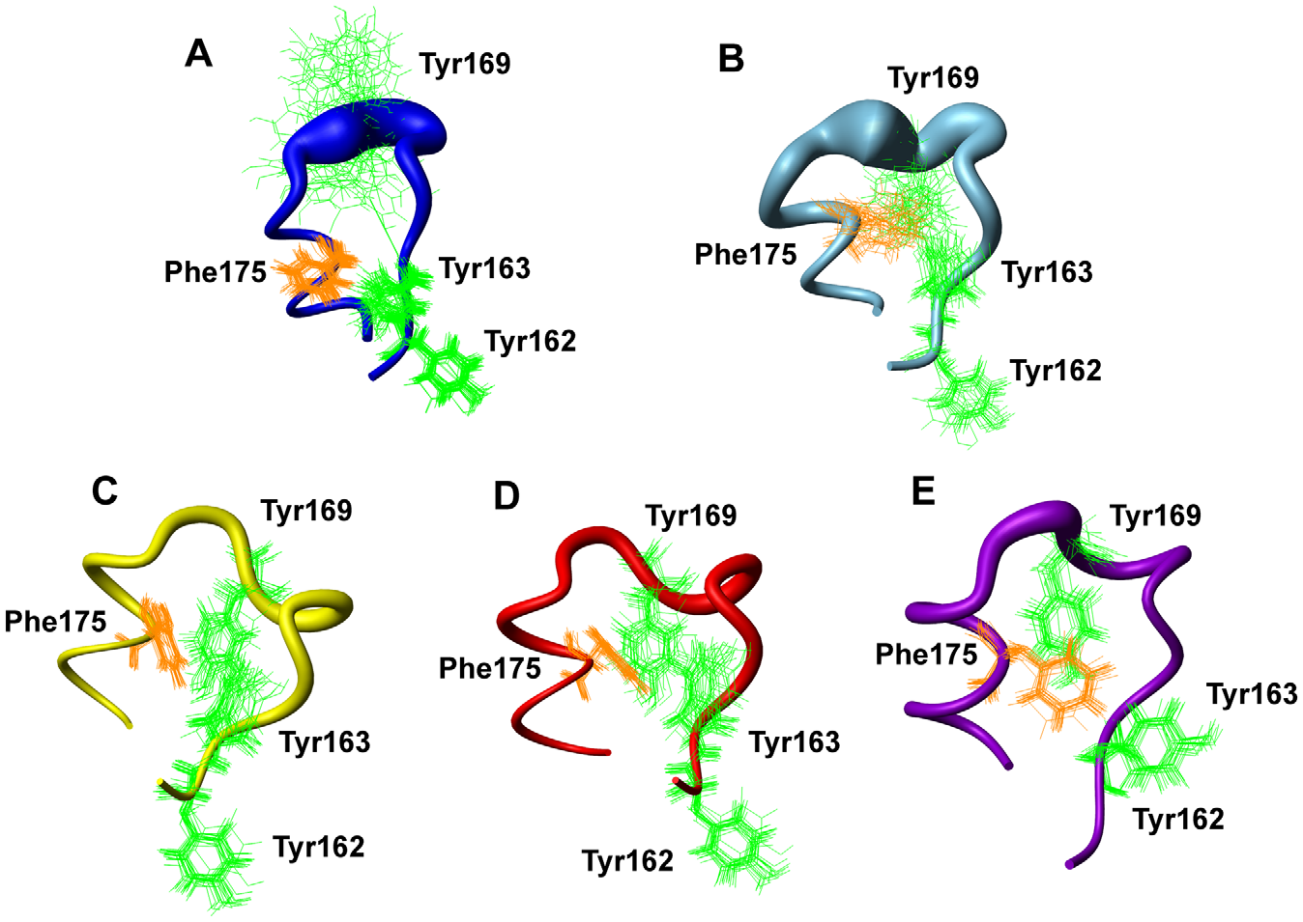
Comparison of local structural variations of different mammalian PrP^C^ from residue Val161 to Cys179 representing β_2_–α_2_ loop. (A) HuPrP(90–231, M129, Q212P) (pdb id 2KUN, this work). (B) WT HuPrP(90–231, M129) (pdb id 1QM1) [25]. (C) Elk PrP (pdb id 1XYW) [34]. (D) Bank vole PrP (pdb id 2K56) [33]. (E) Tammar wallaby PrP (pdb id 2KFL) [32].

One important structural feature of Q212P mutant as opposed to the WT consists in a different mutual orientation of aromatic residues in β_2_–α_2_ loop (Figures 5A and B). Aromatic residues Tyr162, Tyr163, Tyr169 and Phe175 are in close contact in the WT protein thus forming a hydrophobic cluster at the interface of β_2_, α_2_ and α_3_ structural elements [25]. Within β_2_–α_2_ loop region Tyr169, given its central position, interacts with Tyr163 and Phe175 in the WT protein (Figure 5B). In contrast, in Q212P mutant Tyr169 is exposed to solvent resulting highly flexible (Figures 5A). Different orientation of Tyr169 is coupled with a marked twist of Phe175 away from the β_2_–α_2_ loop. The numerous NOE-based distance restraints demonstrate that the aromatic ring of Phe175 is well defined. As a result the whole hydrophobic cluster is changed and shows increased exposure of hydrophobic surface of the protein to solvent (Figure 5A). Interestingly, the β_2_–α_2_ loop regions in PrP structures of elk, bank vole and tammar wallaby display high structural definition (Figure 5). This loop plasticity may therefore modulate the susceptibility of a given species to prion disease [32], [33], [34].

### Mutual orientation of α_2_ and α_3_ helices

**Figure 6:**
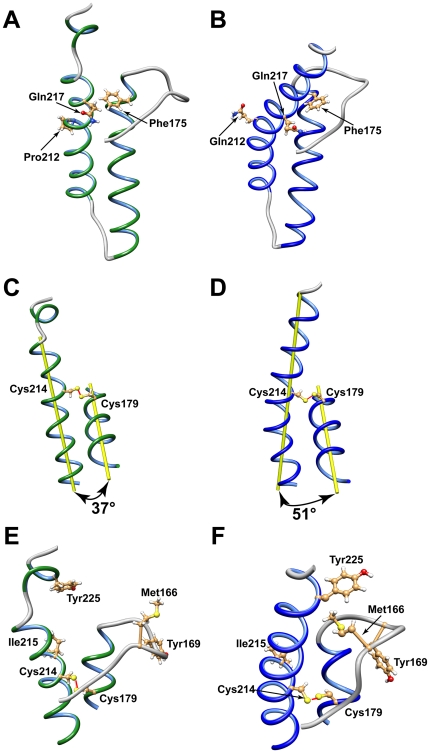
Structural details of HuPrP(90–231, M129, Q212P) (A, C, E) and WT protein (B, D, F). (A) Carton presentation of α_2_, α_3_ and α_4_ helices with mutual orientation of Phe175 and Gln217 in the Q212P mutant. (B) Carton presentation of α_2_ and α_3_ helices with mutual orientation of Phe175 and Gln217 in the WT protein. (C) The mutual orientation of α_2_ and α_3_ helices with indicated inter-helical angle in Q212P mutant. (D) The mutual orientation of α_2_ and α_3_ helices with indicated inter-helical angle in WT protein. (E) Structural organization of β_2_-α_2_ loop and α_3_ and α_4_ helices in Q212P mutant. (F) Structural organization of β_2_-α_2_ loop and α_3_ helix in WT protein.

The disulfide bridge involving Cys179 and Cys214 determines the overall structure of the PrP by fixing the mutual orientation of α_2_ and α_3_ helices. Upon mutation at position 212 the local topology of α_3_ helix remained unchanged although Pro is a well known helix-breaker [42]. However, detailed structural analysis of the Q212P mutant has revealed that α_3_ helix exhibits a small rotation along the helical axis compared to the WT protein. A turn of α_3_ helix around Pro212 is altered to accommodate unfavorable steric interactions of proline with the preceding residue Glu211. The relative orientation of α_3_ helix in comparison to the other secondary structure elements has changed (Figures 6A and 6B). Our experimental data yielded 59 long-range distance restraints, which enabled us to determine the mutual orientation of α_2_ and α_3_ helices with high accuracy. The large number of restraints evenly distributed along the inter-helical surface demonstrated that the C-terminal part of α_3_ helix formed close contacts with the N-terminal part of α_2_ helix (Figure 6A). An illustration of this long-range interaction is the distance between C_ζ_ atom of Phe175 and C_γ_ atom of Gln217, which is 4.9 Å. The corresponding distance in the structure of WT protein is 8.5 Å (Figure 6B). The inter-helical angle between α_2_ and α_3_ helices is 33° in the mutant in comparison to 51° in the WT protein structure (Figures 6C and D). Simultaneously, the distance between helical axes differs by 1.4 Å (Table 2).

**Table 2:** Inter-helical angles and distances between helices α_2_ and α_3_ in high-resolution structures of PrP^C^ proteins a.

	pdb id	Angle (°)	Distance (Å)
HuPrP(90–231, M129, Q212P)	2KUN	33.1	8.4
HuPrP(90-231, M129)	1QM1 [25]	50.9	7.0
Elk PrP^C^	1XYW [34]	47.9	7.7
Bank vole PrP^C^	2K56 [33]	45.6	8.0
Tammar wallaby PrP^C^	2KFL [32]	52.6	6.9

a Calculated with Chimera system [59].

In principle, a reliable source of experimental data on relative orientation of two helices can be obtained by acquiring Residual Dipolar Couplings (RDC) [43]. Our attempts to prepare an NMR sample of HuPrP(90–231, M129, Q212P) protein in a stretched gel though, was unsuccessful due to protein aggregation. Prion protein exhibits a large hydrophobic surface, which is exposed to solution and can therefore unspecifically interact with the medium. In addition, we tried to extract RDC values from NMR IPAP experiments performed at different magnetic fields [44]. Unfortunately, RDC values obtained for the mutant on the basis of self-orientation were too small to be used in structure calculations. Nevertheless, inter-helical angles could be determined with high accuracy without the use of RDC data [45]. In the Q212P mutant protein, the high number of long-range NOE contacts between amino acid residues in α_2_ and α_3_ helices provided sufficient experimental data for defining mutual orientation of the two helices. On average we observed 6 to 8 long-range distance restrains per residue at the inter-helical interface (Figure 4).

## Discussion

**Figure 7:**
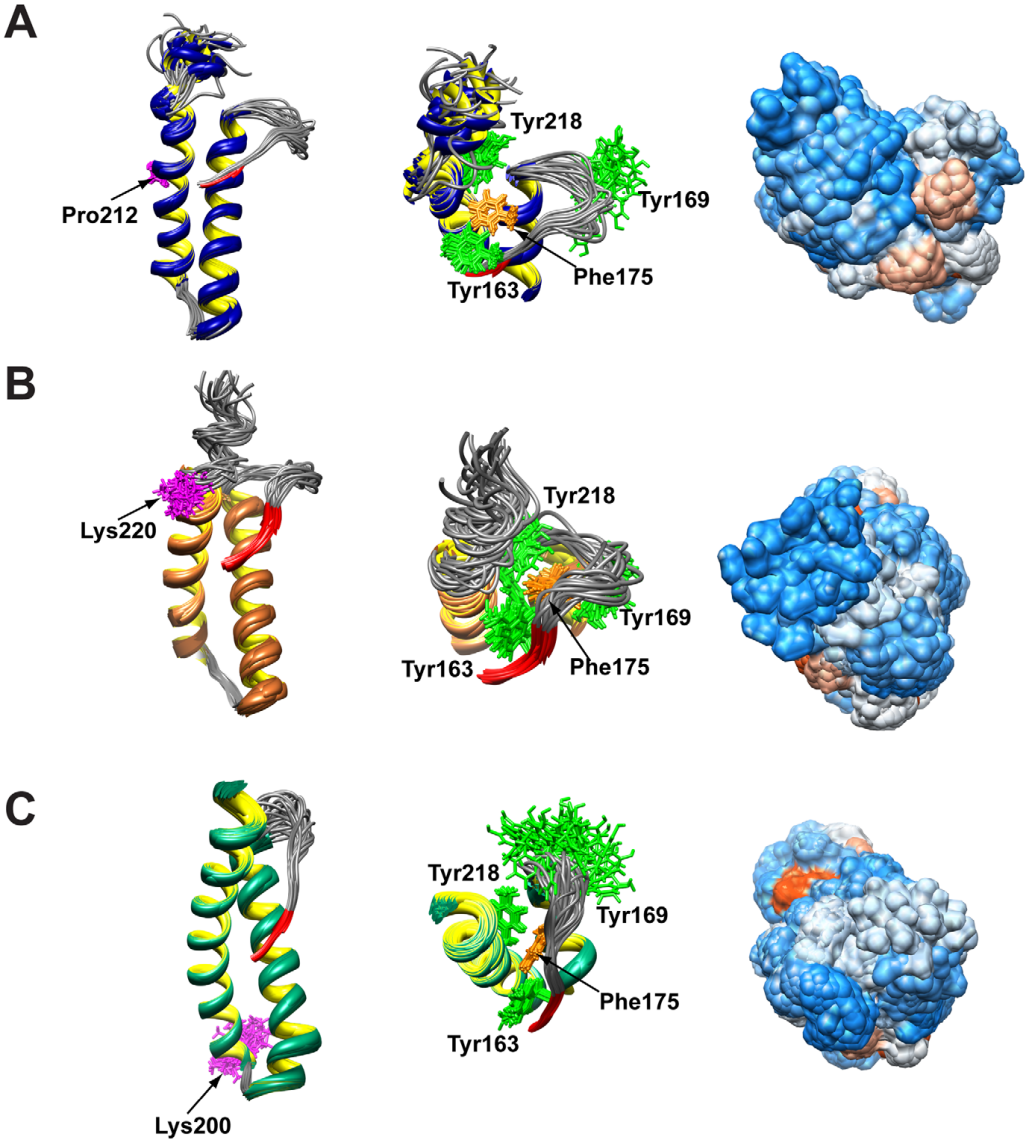
Structural comparison of HuPrP mutants. (A) Structural details of β_2_α_2_α_3_α_4_ region (161–228) of 20 lowest energy structures for HuPrP(90–231, M129, Q212P) mutant. (B) Structural details of β_2_α_2_α_3_ region (161–228) of 20 lowest energy structures for HuPrP(90–231, M129, R220K) (pdb id 1E1U) mutant [31]. (C) Structural details of β_2_α_2_α_3_ region (161–228) of 20 lowest energy structures for HuPrP(90–231, M129, E200K) (pdb id 1FO7) mutant [28]. In all three panels the point mutation is indicated in magenta (left). Top view of the hydrophobic core composed of aromatic amino acid residues (center). Hydrophobicity surface is presented on the right, where red color indicates hydrophobic and blue color represents hydrophilic surface.

In this study we determined the structure of the HuPrP(90-231, M129) carrying the Q212P mutation associated to a GSS syndrome. The high-resolution NMR structure of Q212P mutant revealed unique structural features compared to the WT protein. The most remarkable differences involve the C-terminal end of the protein and the β_2_–α_2_ loop region. The structure of Q212P mutant is the first known example of PrP structure where the α_3_ helix between Glu200 and Tyr226 is broken into two helices. It is noteworthy that a break occurs almost two helical turns beyond Pro212, which is the position of mutation. The break results in dramatic changes in hydrophobic interactions between α_3_ helix and β_2_–α_2_ loop region. In the WT protein long-range interactions between Tyr225 and Met166 define the position of β_2_–α_2_ loop, and thus tertiary structure of the protein (Figure 6F). The distance between C_α_ atoms of Tyr 225 and Met166 in structures of prion proteins of different mammals is typically 8.4 Å, whereas it is 16.5 Å in the Q212P mutant (Table 3). Longer distance is correlated with a marked twist of Tyr225 away from β_2_–α_2_ loop (Figure 6E). Tyr225 in Q212P mutant forms hydrophobic interactions with residues in α_3_ helix (e.g. Ile215, Table 3). These interactions define mutual orientation of α_3_ and α_4_ helices. As Tyr225 is unable to form contact with Met166, hydrophobic cluster is opened and accessible to solvent. Exposure of hydrophobic surface is tightly correlated with orientation of aromatic residues Tyr163, Tyr169 and Phe175 in Q212P mutant (Figure 7A). The opened cleft has been proposed as the binding site for a hypothetical facilitator of prion conversion that may play a role in pathogenic PrP^Sc^ formation [23], [24]. In the WT protein, the solvent exposed surface of β_2_–α_2_ loop and α_3_ helix region is smaller and Tyr169 is buried inside the hydrophobic cluster.

**Table 3:** Distances between Tyr225 and residues within β_2_–α_2_ loop region and α_3_ helix.

PrP^C^	Y169–Y225 a,b	M166–Y225 a,c	I215–Y225 a,d
HuPrP(90–231)	14.1±1.2	8.4±0.6	19.2±0.6
HuPrP(90–231, Q212P)	18.8±2.0	16.5±1.6	9.9±1.1
HuPrP(90–231, E200K)	11.7±1.4	8.4±0.5	19.2±0.5
HuPrP(90–231, R220K)	13.3±0.9	8.4±0.5	19.9±1.0

a All presented data are average distances and standard deviations in Å calculated for ensemble of 20 structures deposited in pdb.

b Distances between carbons Tyr169 C_α_ and Tyr225 C_α_.

c Distances between carbons Met166 C_α_ and Tyr225 C_α_.

d Distances between carbons Ile215 C_δ1_ and Tyr225 C_z_.

The increased flexibility in the C-terminal part of α_3_ helix in PrP mutants has been described before [31], [46]. In the HuPrP(90–231, M129, R220K) artificial mutant for example, α_3_ helix is well ordered up to the point mutation (Figure 7B) [31]. After this mutation, α_3_ helix shows increased flexibility and is less ordered. At the same time, R220K mutation has not altered the hydrophobic interactions between aromatic residues of β_2_–α_2_ loop and α_3_ helix (Figure 7B and Table 3). On the other hand, the structure of CJD-related mutant HuPrP(90-231, M129, E200K) has revealed features in β_2_–α_2_ loop region similar to the Q212P mutant (Figure 7C) [28]. In E200K variant, Tyr169 is exposed to solvent and shows increased flexibility. The remaining aromatic residues in β_2_–α_2_ loop (Tyr163 and Phe175) form a hydrophobic cluster through interaction with Tyr218 and Tyr225 in α_3_ helix (Figures 7C). The effects of both Q212P and R220K mutations in terms of unstructured C-terminal parts of proteins are comparable (Figures 7A and B). In support, recently published crystal structures of the pathological HuPrP(90–231, D178N, M/V129) and HuPrP(90–231, F198S, M/V129) mutants demonstrated similar orientations of Tyr169 outside the globular part [46].

Mutations in HuPrP segregating with familial TSE may provoke reduced stability in the structure of the PrP^C^ form. This event could enhance the tendency of PrP^C^ to adopt different conformational states, some of which would then lead to the conversion into the PrP^Sc^ isoform [41]. It is noteworthy that HuPrP carrying either D178N or F198S mutations actually showed a reduced thermodynamic stability compared to the WT HuPrP [16].

The biological implications of our findings could provide new clues to our understanding of the structural changes occurring in PrP^C^ during prion formation. Special interest in prion biology is focused on the epitope formed by the β_2_–α_2_ loop and the α_3_ helix, because it seems to modulate specific intermolecular contacts involved in the development of TSE. In the case of Q212P mutant it is possible to argue that the larger solvent exposure of this epitope causes an altered interaction with other, yet unknown, cellular cofactors, chaperones, or PrP^C^ ligands. Recent NMR studies provide evidences that some GSS mutations increase the affinity of the PrP for binding lipid structures [47]. The altered conformation observed in the Q212P mutant might cause a different affinity for cellular membranes and, consequently, an aberrant localization of PrP in the cell compartments, to favor formation of altered ER topologies [20], [21]. Independent evidences derived from cell culture expressing the mutant Q212P showed that this point mutation affects folding and maturation of PrP^C^ in the secretory pathway of neuronal cells [19], [22]. These authors investigated the generation and the turnover of Q212P and others mutants in mouse neuroblastoma N2a cells discovering an intracellular post ER control pathway that selectively routes aberrant PrP species to the lysosomes [48]. The structure-function relationship suggested with our work could provide a biological basis for understanding the spontaneous generation of PrP^Sc^ in inherited prion disease.

## Materials and Methods

### Plasmid Construction

The Q212P mutant was constructed using the QuikChange™ kit (Stratagene) utilizing primers 5′-CGC GTG GTT GAG CCG ATG TGT ATC ACC C-3′ and 5′- GGG TGA TAC ACA TCG GCT CAA CCA CGC G -3′ and HuPrP(90-231, M129) as template. The DNA product was then inserted into a pET-11a vector (Novagen) containing a His-tag sequence at the carboxy-terminus of the inserted sequence. The cloned DNA sequences were verified by sequencing.

### Protein Expression and Purification

Freshly transformed overnight culture of *E. coli* BL21 (DE3) cells (Stratagene) was added at 37°C to 2 L of minimal medium (MM) plus ampicillin (100 µg/mL). For isotope labeling 4 g/L [^13^C_6_] glucose and 1 g/L [^15^N] ammonium chloride were added. At 0.8 OD_600_ expression was induced with isopropyl β-D-galactopyranoside to a final concentration of 0.8 mM. The cells were harvested 12 h after induction. The cells were lysed by a French press (EmulsiFlex-C3) and the inclusion bodies were washed in buffer containing 25 mM Tris-HCl, 5 mM EDTA, 0.8% TritonX100, pH 8, and then in bi-distilled water several times. Pure inclusion bodies were solubilized in 5 volumes of 6 M GndHCl, loaded onto a 5 mL HisTrap column (GE Healthcare) equilibrated in binding buffer (2 M GndHCl, 500 mM NaCl, 20 mM TrisHCl, 20 mM imidazole, pH 8) and eluted with 500 mM imidazole. Subsequently, the protein was purified by reverse-phase (Jupiter C4, 250 mm×21.2 mm, 300 Å pore size, Phenomenex) and separated using a gradient of 0-95% acetonitrile and 0.1% trifluoroacetic acid. The purified protein was lyophilized and dissolved in 8 M GndHCl. Refolding was performed by dialysis against refolding buffer (20 mM sodium acetate-d_3_, 0.005% NaN_3_, pH 5.5) using a Spectrapor-membrane (MW 3000). Purified protein was analyzed by SDS-polyacrylamide gel electrophoresis under reducing condition, western blot, and electrospray mass spectroscopy.

### NMR spectroscopy

All NMR experiments used for structure calculation were performed on ^13^C, ^15^N double labeled HuPrP(90-231, M129, Q212P) protein on Varian VNMRS 800 MHz NMR spectrometer using triple ^1^H/^13^C/^15^N resonance cold probe-head with inverse detection at 298 K. NMR sample consisted in a 0.8 mM concentration of ^13^C, ^15^N-labeled protein in sodium acetate buffer. NMR experiments with detection of HN were performed in 90%/10% H_2_O/^2^H_2_O, pH 5.5. HC detected NMR datasets were acquired in 100% deuterated buffer. Relaxation measurements including ^15^N longitudinal (*R*_1_), transversal (*R*_2_) relaxation rates and {^1^H}-^15^N NOE were performed on two magnetic fields of 11.7 T and 16.4 T. Standard triple resonance NMR experiments were used for assignments and to obtain distance restraints. Chemical shifts were referenced considering external DSS. All recorded spectra were processed by NMRPipe software [49] and analyzed with Sparky [50] and CARA software (available for free download from http://www.nmr.ch) [51].

### Structure calculations

The initial structure calculations were done by program CYANA 2.1 [52]. The automatic NOE assignment procedure [53] yielded 2205 distance constraints which were imported into CNS (version 1.2) software [54], [55] for structural refinement. High resolution 3D structure of Q212P mutant was determined based on 1244 intra- and sequential, 491 short-range and 470 long-range distance constraints supported by 188 backbone torsion angle restraints (Table 1). Finally, the structure refinement was performed using explicit solvent model in YASARA program suite (www.yasara.org) [56]. The final ensemble of 20 lowest energy structures exhibited good convergence and very high definition. Validation procedure using PROCHECK-NMR [57] and WhatIF [58] programs demonstrated that the final family of 3D structures agreed with the distance restrains and offered good geometry and side chain packing.
